# Comparative analysis of the oral microbiota between iron-deficiency anaemia (IDA) patients and healthy individuals by high-throughput sequencing

**DOI:** 10.1186/s12903-019-0947-6

**Published:** 2019-11-21

**Authors:** Ranhui Xi, Renke Wang, Yuan Wang, Zhenting Xiang, Zhifei Su, Zaiqiang Cao, Xin Xu, Xin Zheng, Jiyao Li

**Affiliations:** State Key Laboratory of Oral Diseases & National Clinical Research Center for Oral Diseases, Department of Cariology and Endodontics, West China Hospital of Stomatology, Sichuan University, Chengdu, 610041 China

**Keywords:** Oral microbiota, Iron-deficiency anaemia, Infective endocarditis, High-throughput sequencing

## Abstract

**Background:**

The relationship between oral microbiota and IE (infective endocarditis) is well established. Opportunistic pathogens in normal oral flora enter the bloodstream through daily oral cleaning or invasive dental procedures, leading to the occurrence of infective endocarditis. An in vitro iron-deficient condition leads to a drastic community shift in oral microbiota with increasing proportions of taxa related to infective endocarditis. To investigate the relationship among insufficient iron supply, oral microbiota and the risk of IE and to conduct a population amplification study, iron-deficiency anaemia is used as an in vivo model.

**Methods:**

This cross-sectional study enrolled 24 primary iron-deficiency anemia (IDA) patients from 2015.6 to 2016.6 from the hematology department of West China Hospital, Sichuan University, and 24 healthy controls. High-throughput sequencing compared the dental plaque microbiota of 24 IDA (iron-deficiency anaemia) patients and 24 healthy controls.

**Results:**

Sequences were classified into 12 phyla, 28 classes, 50 orders, 161 genera and 497 OTUs (the IDA and control groups shared the same 384 OTUs). Iron deficiency leads to lower internal diversity in the oral flora. The abundances of genera Corynebacterium, Neisseria, Cardiobacterium, Capnocytophaga, and Aggregatibacter were significantly higher in healthy controls, while genera Lactococcus, Enterococcus, Lactobacillus, Pseudomonas and Moraxella showed higher proportions in the IDA group (*P* < 0.05). The relative abundances of genera Lactococcus, Enterococcus, Pseudomonas and Moraxella were significantly negatively correlated with the concentration of serum ferritin (*P* < 0.05).

**Conclusions:**

Without an increase of oral streptococci, the main pathogen of IE, it is difficult to determine whether IDA can increase the risk of IE. However, the iron-deficient condition did lead to changes in the oral microbiota community structure. The genera that showed higher proportions in the IDA group were frequently reported as antibiotic-resistant. As antibiotics are commonly recommended to prevent IE before dental procedures, this study offers new ideas of personalized prevention of IE.

## Background

Infective endocarditis (IE) is an infectious disease of the inner lining of the heart, the cardiac valves and the endocardium with high morbidity and mortality. Oral bacteria are closely related to IE and have attracted close attention from cardiologists, dentists, and patients. IE is caused by a variety of infectious agents, ranging from oral streptococci (30–60% of detection rate in blood cultures or endocardial vegetation) to *Staphylococcus aureus*, enterococci and so on [1, 2]. For decades, hundreds of case reports and correlation analyses have revealed that oral infection and dental operations (tooth extraction, dental cleaning, implantation, root canal therapy, etc.) can lead to the occurrence of infective endocarditis [3, 4]. As a part of dental plaque, oral bacteria can enter the bloodstream, causing low-grade but frequent bacteremia through daily habits, such as chewing or tooth brushing [5, 6]. The 2015 ESC Guidelines for the management of infective endocarditis, which was published by the European Society of Cardiology (ESC), classified the situations with risk into two categories: dental procedures and other at-risk procedures. The guidelines emphasized that strict dental hygiene and twice-a-year dental follow-ups are the first preventive measures to be followed by the high-risk population. Moreover, for the high-risk population, the first recommendation for prophylaxis is antibiotic prophylaxis before dental procedures, such as endodontic treatment, periodontal treatment, invasive treatment of the oral mucosa, tooth extraction, tooth implantation and so on [7, 8]. However, no research has focused on the relationship between oral microecological changes and infective endocarditis.

In our previous study to examine the influence of elemental iron on the structure of human salivary microbiota, a drastic community shift was observed. The saliva of 6 healthy individuals was cultured separately with SHI medium, and the iron-deficient environment was created by the addition of the iron chelator Bipy (2,2 ‘-bipyridyl). Under iron-depleted conditions, the proportion of strains implicated in IE, such as α-haemolytic *Gemella* spp., *Streptococcus* spp. and *Granulicatella* spp*.*, increased dramatically with stronger haemolytic ability, and this change could partly be reversed by the addition of free Fe^2+^ [9].

Iron is a significant mineral element for most microbes and the host itself. Elemental iron participates in basic biochemical processes, such as electron transfer, redox reactions, ATP synthesis, the TCA (tricarboxylic acid cycle), cell aerobic and anaerobic respiration, gene regulation and DNA biosynthesis [10]. Although iron is one of the most abundant elements in the earth’s crust, it is not readily available for colonizing bacteria due to its low solubility in the human body. Hosts and microbes compete fiercely for iron. The human body produces extracellular iron-chelating glycoproteins, such as transferrin, ferritin, and lactoferrin, along with intracellular ferritin and haem to obtain elemental iron [11, 12]. In physiological conditions, the host inhibits the growth and reproduction of microorganisms by limiting the iron supply, which plays an important role in the host immune defence. Successfully colonized oral bacteria obtain iron in various ways to compete with each other as well as with the host. Oral streptococci and spirochetes (*Treponema denticola*, *Treponema vincentii* and *Treponema socranskii*) destroy erythrocytes and muscle cells to obtain intracellular iron [11, 12]. *Actinobacillus actinomycetemcomitans* can produce a variety of ferritin isoforms to chelate the ferric ions in host proteins [13]. *Campylobacter rectus* utilizes the iron in transferrin [14], while haem is the main iron source for *Porphyromonas gingivalis* [15, 16]. Other microorganisms unable to utilize extracellular iron, such as *Legionella pneumophila* and *Mycobacterium tuberculosis*, are limited to obligatory intracellular organisms [17]. In addition, *Bifidobacterium* has very little demand for iron, while in *Lactobacillus*, manganese ions are capable of replacing iron ions in some metalloenzymes, covering almost all basic functions that iron usually accomplishes [18].

The mouth harbours over 700 species of bacteria. Oral microorganisms mainly exist in the form of a biofilm, maintaining a dynamic ecological balance with the host in a physiological state. The disruption of this ecological balance is necessarily related to oral infectious diseases (chronic periodontitis, dental caries, periapical periodontitis, pericoronitis) as well as systemic conditions, such as cardiovascular diseases, diabetes mellitus, preterm birth and cancer [19]. In view of the important relationship between oral bacteria and infective endocarditis combined with the previous research finding that iron-deficiency conditions in vitro shifted the ecological balance to specific pathogenic bacteria, we speculated that the iron-deficient environment in the human mouth may lead to changes in the bacterial community and a higher risk of infective endocarditis.

However, there are definite differences between in vivo growth and in vitro cultivation. Considering the influence of various factors in the human oral cavity, it is still uncertain whether the same community shift would exist. Furthermore, we chose the iron-deficiency anaemia (IDA) population to be the in vivo model of iron depletion in human oral microbiota. IDA is the most common form of anaemia and is caused by an inadequate dietary intake of elemental iron, iron malabsorption, iron loss due to menstruation, haemorrhage of the digestive tract, infection, etc. [19]. In 2010, the WHO reported that more than 1.2 billion people all over the world were affected by IDA [20]. The concentrations of haemoglobin, ferritin, and transferrin in the blood of IDA patients were significantly reduced, and the intracellular storage of ferritin was reduced to less than half that of healthy individuals [21, 22]. The levels of free iron and iron sources (transferrin, lactoferrin, and ferritin) in the oral cavity also decreased significantly [23–26].

Moreover, the oral cavity is a complex environment, and less than half of the bacterial species can be cultivated. High-throughput sequencing of 16S rRNA genes provided more comprehensive information of the complex microbiota populations due to its deep coverage depth and multiple output data, which can detect species with low DNA content and unculturable taxa.

In general, this study chose IDA patients as an in vivo model to investigate the relationship among insufficient iron supply, oral microbiota and the risk of IE by comparing the oral microbiota community profile between healthy and IDA populations with high-throughput sequencing. The results of this study may help guide oral hygiene practices and clinic oral operation for IDA populations and offer personalized prevention for IE.

## Methods

### Study design

From June 2015 to June 2016, after the stringent screening,24 IDA patients (male = 2) and 24 healthy volunteers (male = 2) were recruited into this cross-sectional study. Authorization of the study was given by the ethics committee of the West China Hospital of Stomatology, Sichuan University. Oral examination was performed by the same dentist and hematological examination was carried out by the clinical laboratory of West China hospital. To take part in this research, all 48 study participants signed a voluntary written consent form.

### Participants

All patients in the IDA group in the study were diagnosed with IDA according to WHO diagnosis standards before any treatment from the department of haematology of West China Hospital, Sichuan University, China. The preliminary screening included 40 patients, and 16 of whom were excluded mainly result from dental caries and periodontal diseases (exclusion criteria are shown in Table 1). Another 24 healthy volunteers were recruited from local residents of Sichuan Province. The IDA patients and controls were matched by age and sex. The IDA and control groups in this study were both free from systemic and oral infective diseases to avoid undesired effects on the oral micro-ecosystem.

**Table 1:** Exclusion criteria

Exclusion criteria	
1. Under eighteen-years-old 2. Systemic disease (malignant tumour, alimentary tract haemorrhage, systemic infection) 3. Pregnant or breastfeeding women 4. Active dental caries, apical periodontitis, periodontal diseases, pericoronitis 5. Orthotics or removable denture in the mouth 6. Less than 24 teeth in the mouth 7. Systematic antibiotics or immunomodulators within 3 months 8. Oral topical administration of antibiotics or fluorine within 2 weeks	

### Sampling

Sampling was conducted according to the Manual of Procedures for Human Microbiome Project Core Microbiome Sampling Protocol by the NIH. Participants were instructed to refrain from eating or brushing their teeth for 3 h. Supragingival dental plaque was collected from multiple teeth (21 41 24 44 16 36) with a Gracey scaler by scraping the tooth surface [27]. Samples were placed in 1 ml PBS buffer immediately and then transferred on ice within 30 min to the laboratory.

### DNA extraction

The QIAamp DNA Mini Kit (Qiagen, USA) was used to extract the total bacterial genomic DNA from the plaque samples, with an added treatment of lysozyme for bacterial lysis (2.5 mg/ ml, Sigma, USA). The determination of DNA purity and concentration was carried out by a NanoDrop ND2000 spectrometer (Thermo Fisher Scientific, USA).

### PCR amplification

Bacterial 16 SRNA was polymerized using common primers targeting the v3-v4 region of bacterial 16S rRNA (338F 5 ‘-barcode-actcctacg-ggaggcagcag-3’ and 806R 5 ‘-actcctacgggaggcagcag-3’), where the barcode is an eight-base sequence unique to each sample. Reactions were carried out with template DNA (1 μL, 10 ng), each primer (5 mM, 1 μL) and PrimeSTAR® Max DNA Polymerase (15 μL, Takara, Japan) with the following amplification procedure: 3 min at 98 °C, 28 cycles (30 s at 98 °C, 45 s at 56 °C, 30 s at 70 °C) and a final 5 min at 70 °C. Amplicons were evaluated by agarose gel electrophoresis (1.5%).

### Illumina MiSeq sequencing and analysis

After purification in equimolar concentrations, the amplicons were sequenced on an Illumina MiSeq platform at MajorBio Technology Co. according to the standard protocols. The raw reads were then uploaded to the NCBI Sequence Read Archive (SRA) database (SRA number: SUB4333399).

Sequences were filtered with QIIME (version 1.9.1), and those shorter than 150 bp or those containing more than 2 nucleotide mismatches or ambiguous characters were removed. Reads were then clustered to operational taxonomical units (OTUs) with 97% similarity with the use of USEARCH (version 7.0). Each taxon was classified according to the human oral microbiome database (HOMD) by the QIIME platform and RDP Classifier (version 2.2) with a confidence threshold of 70%.

Alpha diversity analysis, including Chao, ACE, Shannon, Simpson, and evenness indices and rarefaction curves, were carried out with MOTHUR (version v.1.30.1). The clustering analysis of the evolution of the microbiotas was calculated by QIIME, and PCoA was calculated by R (version 3.2.5). Taxonomy-based analyses and identification of phylotypes that were significantly different among groups were carried out with the Naïve Bayesian Classifier and MOTHUR, respectively.

LEfSe was used to discover the microorganismal biomarker [28, 29], while other statistical analyses were carried out by SPSS (version 16.0). The level of statistical significance was accepted as *P* < 0.05. All tests for significance were two-sided.

## Results

### Participants

The IDA group consisted of 24 patients diagnosed with IDA (serum ferritin< 12; Hb < 110; MCV < 80) before treatment and without any other oral or systemic disease. Supragingival dental plaque was collected from the IDA group (male = 2) and 24 healthy volunteers (male = 2) matched by sex. The participants were all Han Chinese Sichuan residents. There was no significant difference in age distribution between the two groups (*P* = 0.337) (Table 2). All samples were collected from September to December 2015. The IDA group was then separated into IDA_s (severe anaemia, *n* = 8) and IDA_m (moderate anaemia, *n* = 16) (Additional file 1: Table S1), following the WHO classification criteria of IDA [20].

**Table 2:** Clinical features of the participant population

Variable (average)	IDA group(*n* = 24)		Control group (*n* = 24)		Normal reference value
Age (years)	29.54 ± 9.90	18–47	28.70 ± 6.71	18–40	–
Hg(g/L)	85.70 ± 16.1	58–119	130.6 ± 8.81	113–150	115–150
MCV (fL)	72.43 ± 7.72	60–86.7	88.55 ± 4.96	81.4–98.4	82–100
FRE (ng/L)	4.94 ± 2.15	1.1–11.7	58.14 ± 30.1	29.14–126	24–336

### Taxonomic analysis

A dataset containing 1,033,586 high-quality sequences with a median read length of 445.94 bp was generated by high-throughput sequencing with the Illumina MiSeq platform. A total of 412,348 raw reads and 183,710,717 bp were obtained from the IDA group, while 664,472 reads and 297,113,681 bp were obtained from healthy controls. Reads were clustered into operational taxonomical units (OTUs) with 97% similarity and classified into 12 phyla, 28 classes, 50 families, 161 genera, 313 species, and 516 OTUs according to the human oral microbiome database (HOMD). With the removal of taxa with less than 100 reads, 19 OTUs were excluded from further analysis.

**Fig. 1:**
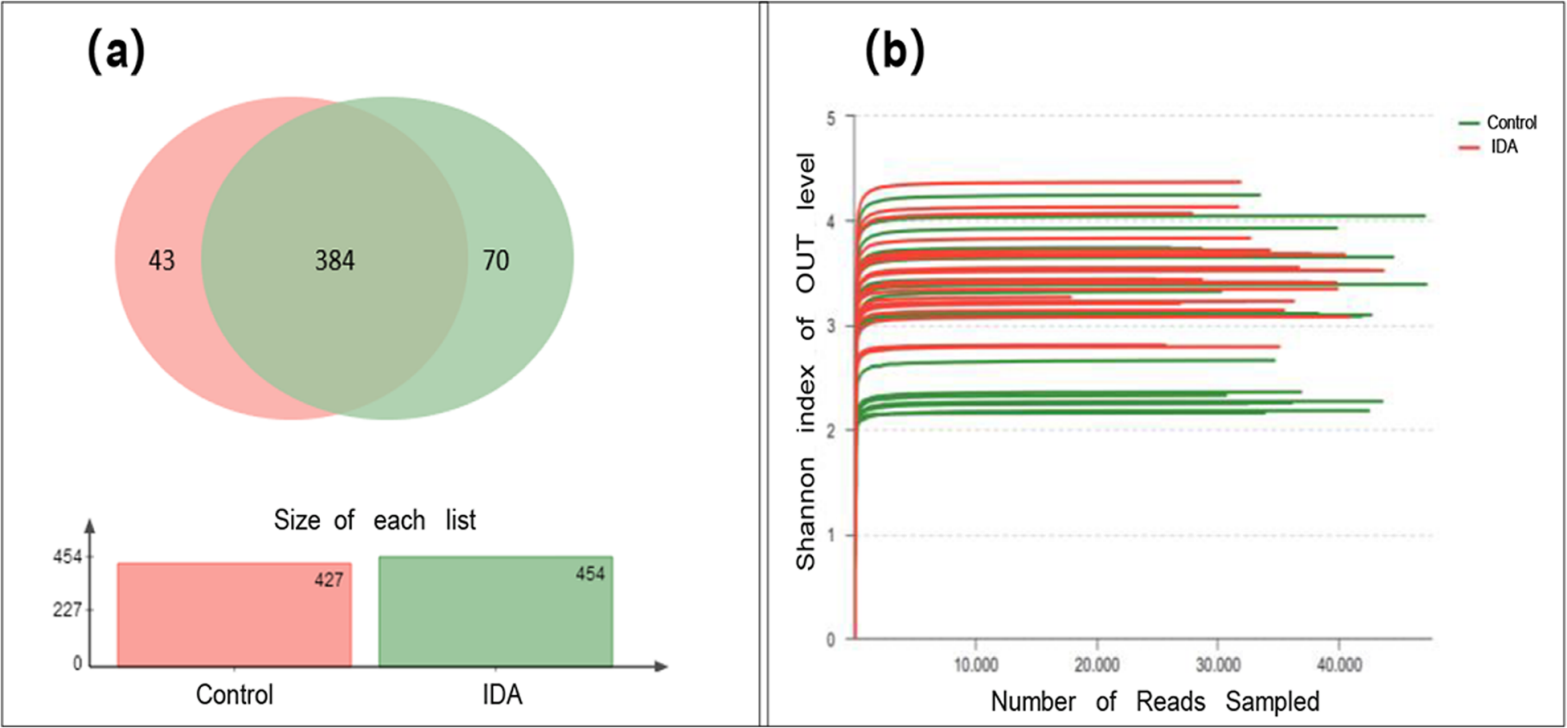
Venn diagram and rarefaction analysis. **a** Venn diagram of different and shared OTUs between the IDA and control groups. **b** Rarefaction analysis of the Sobs index of each sample

According to the Venn diagram of component OTUs (Fig. 1a), sequences of the IDA group showed 454 OTUs, and the healthy controls showed 427 OTUs. The two groups shared more than 90 % of the OTUs (384). Cluster analysis indicated that there were abundant bacterial species in both groups, and IDA did not lead to significant changes in the number of microbial species.

### Richness and diversity analysis

Sobs index was calculated by MOTHUR, and the diagram of the rarefaction curves was prepared by R to evaluate the rationality of sequencing depth and data volume (Fig. 1b). The sequencing data were also analysed with MOTHUR for alpha diversity analysis, including the ACE, Chao1, Shannon, Simpson, Shannoneven and Simpsoneven indices (Table 3).

**Table 3:** Alpha diversity indices

Variable (average)	IDA group(*n* = 24)	Control group(*n* = 24)	*P* value	Q value
Shannon	4.2296 + 0.33429	4.0145 + 0.57635	0.1206	0.1369
Simpson	0.031698 + 0.012867	0.052236 + 0.03746	0.0145^*^	0.02541^*^
Ace	306.57 + 61.323	331.86 + 54.177	0.1369	0.1369
Chao 1	303.12 + 63.103	331.88 + 55.689	0.1009	0.1369
Shannon evenness	0.76831 + 0.035344	0.71767 + 0.086783	0.0111^*^	0.02541^*^
Simpson evenness	0.14538 + 0.040599	0.1086 + 0.057768	0.0141^*^	0.02541^*^

Q value: corrected *P* value; correction method: FDR; ^*^ 0.01 < *P* ≤ 0.05

According to the rarefaction curves, the flat end of the curve indicated that the sequencing data volume is reasonable, and increasing the volume does not lead to significant changes in the number of OTUs. The ACE, Chao1 and Shannon’s indices showed no difference between the two groups (corrected *P* value> 0.05). Moreover, significantly lower Simpson and evenness indices were observed in the IDA group, indicating that the statistically significantly higher alpha diversity of the control group may be related to the uniform distribution of different species of healthy controls.

**Fig. 2:**
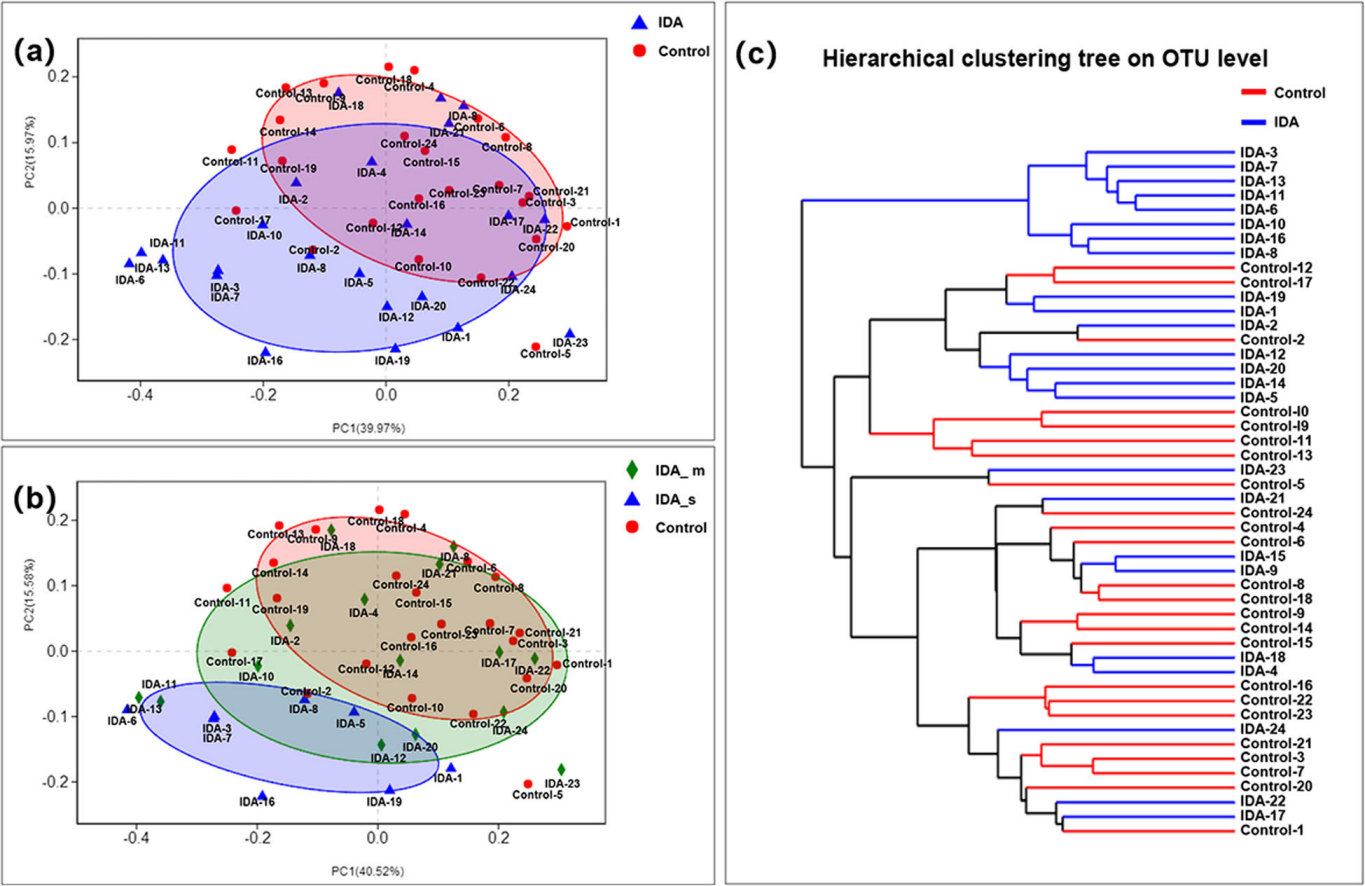
Diversity analysis**a** PCoA of community structures from IDA and control groups. The triangles and circles represent the microbiota of the IDA group and healthy controls, respectively. **b** PCoA of community structures from IDA_s, IDA_m and control groups. The rhombus, triangles, and circles represent the microbiota of IDA_s, IDA_m, and healthy controls, respectively. **c** Hierarchical clustering tree (OTU level)

The degree of similarity between microbial communities was measured by beta diversity analysis. Principal coordinates analysis (PCoA) was performed under a weighted UniFrac scheme based on OTU compositions (Fig. 2a, b). Dissimilarities between community structures were reflected in the coordinate diagram as distances between symbols. In Fig. 2a, the triangles and circles represent the microbiota of the IDA group and healthy controls, respectively. The amounts of the two maximum variations (39.97 and 15.97%) were explained by the values of the abscissa principal coordinate axis 1 (PC1) and ordinate principal coordinate axis 2 (PC2). In Fig. 2b, the rhombus, triangles, and circles represent the microbiota of IDA_s, IDA_m and healthy controls. The amount of the two maximum variations were 40.52 and 15.58%. PCoA could not separate dental plaque samples of IDA populations from healthy controls, while segregation between the IDA_S and control groups was observed. The results showed that the more severe the anaemia, the greater the difference in microecological community structure. Using Bray-Curtis distances, microbiota phylogenetic trees were generated, and each sample was represented by a branch on the tree (Fig. 2c). Although microbiota of the IDA tended to cluster (IDA-3, IDA-7, IDA-13, IDA-11, IDA-6, IDA-10, IDA-16, and IDA-8), the two groups could not be completely separated.

### Community structures

**Fig. 3:**
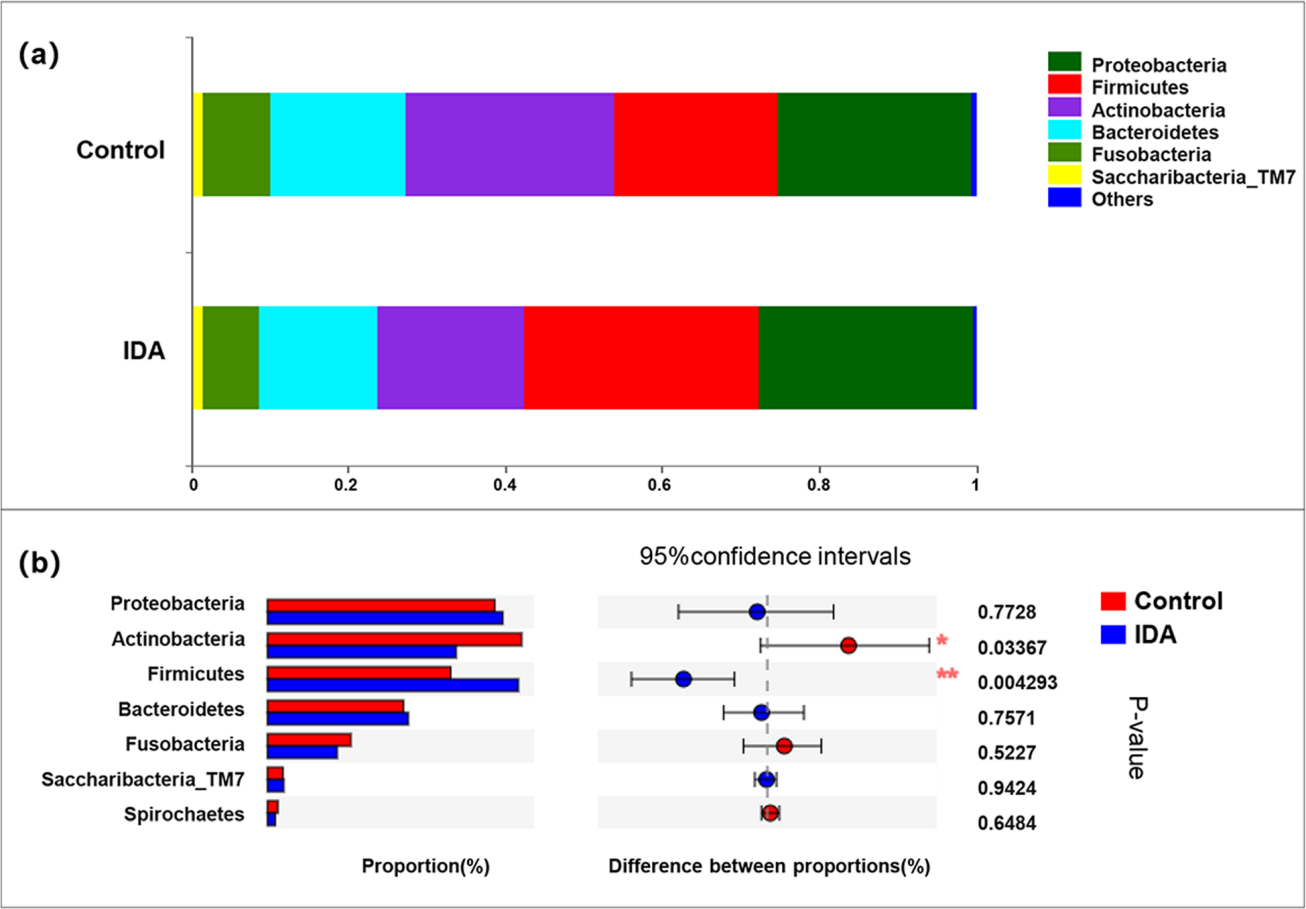
Microbiota structure analysis at phylum level **a** Bar plot of the percent of community abundance. **b** Comparison of bacterial taxonomy (> 1% relative abundance) of samples between healthy controls and IDA patients, * 0.01 < *P* ≤ 0.05,** 0.001 < *P* ≤ 0.01

Although the two groups shared almost the same OTUs, iron deficiency affected the composition of oral microbiota. Twelve phyla were observed in this study, and more than 95% of sequences belonged to seven phyla: Firmicutes (27.52% of OTUs could be classified at the phylum level in the IDA group and 20.11% in the control group), followed by Proteobacteria (25.82 and 24.92%), Actinobacteria (20.69 and 27.88%), Bacteroidetes (15.46 and 14.94%), Fusobacteria (7.65 and 9.14%), Spirochaetes (0.88 and 1.14%), Saccharibacteria_TM7 (1.78 and 1.71%) and others (Fig. 3a).

**Fig. 4:**
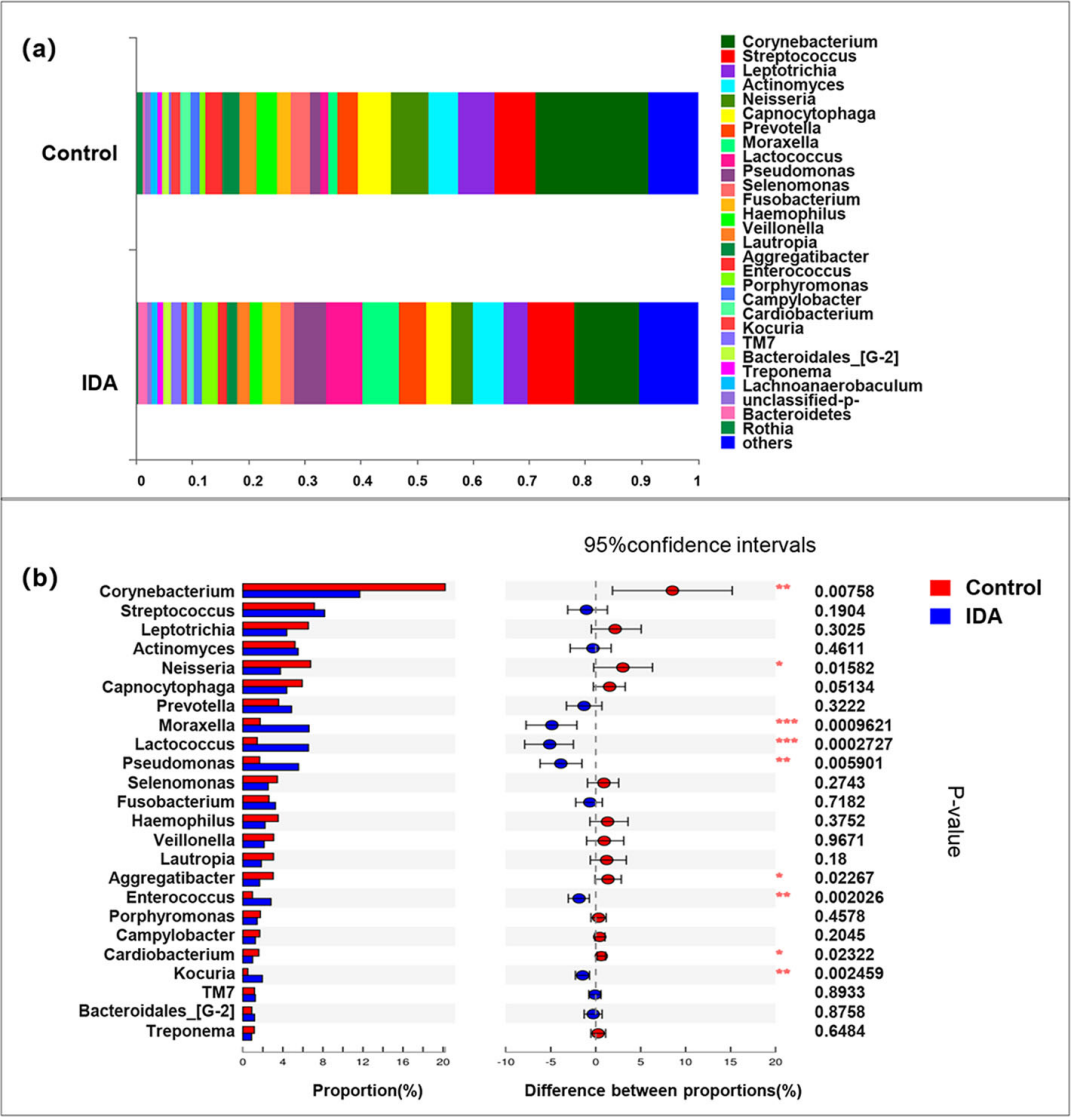
Microbiota structure analysis at genus level **a** Bar plot of the percent of community abundance. **b** Comparison of bacterial taxonomy (> 1% relative abundance) of samples between healthy controls and IDA patients, * 0.01 < *P* ≤ 0.05,** 0.001 < *P* ≤ 0.01

At the genus level, 25 genera were predominant, and these genera accounted for over 93% of the total sequences. The top 8 abundant genera in healthy controls were Corynebacterium (20.21%), Streptococcus (7.141%), Neisseria (6.766%), Leptotrichia (6.542%), Capnocytophaga (5.931%), Actinomyces (5.206%), Prevotella (3.57%), and Haemophilus (3.523%). The predominant genera of IDA patients were Corynebacterium (11.68%), Streptococcus (8.172%), Moraxella (6.605%), Lactococcus (6.549%), Pseudomonas (5.561%), Actinomyces (5.513%), Prevotella (4.868%), and Leptotrichia (4.384%). (Fig. 4a).

Additionally, taxa with significantly different abundances (*P* < 0.05) between the two groups were analysed with the Wilcoxon rank sum test at the phylum and genus levels (two-sided test, confidence interval 0.95).

At the phylum level, Actinobacteria were significantly more abundant, and Firmicutes were significantly less abundant in the healthy oral microbiota (P < 0.05, Fig. 3b). At the genus level, predominant genera such as Streptococcus, Pseudomonas, Enterococcus, Moraxella and Lactococcus were significantly enriched in the IDA group, while Corynebacterium, Neisseria, Capnocytophaga, Cardiobacterium, and Aggregatibacter were more abundant in healthy controls. (*P* < 0.05, Fig. 4b). Notably, the inter-group difference analysis of low-abundance bacteria (relative abundance < 1%) showed that Lactobacillus and Kocuria genera were enriched in the IDA group (Additional file 1: Table S2).

**Fig. 5:**
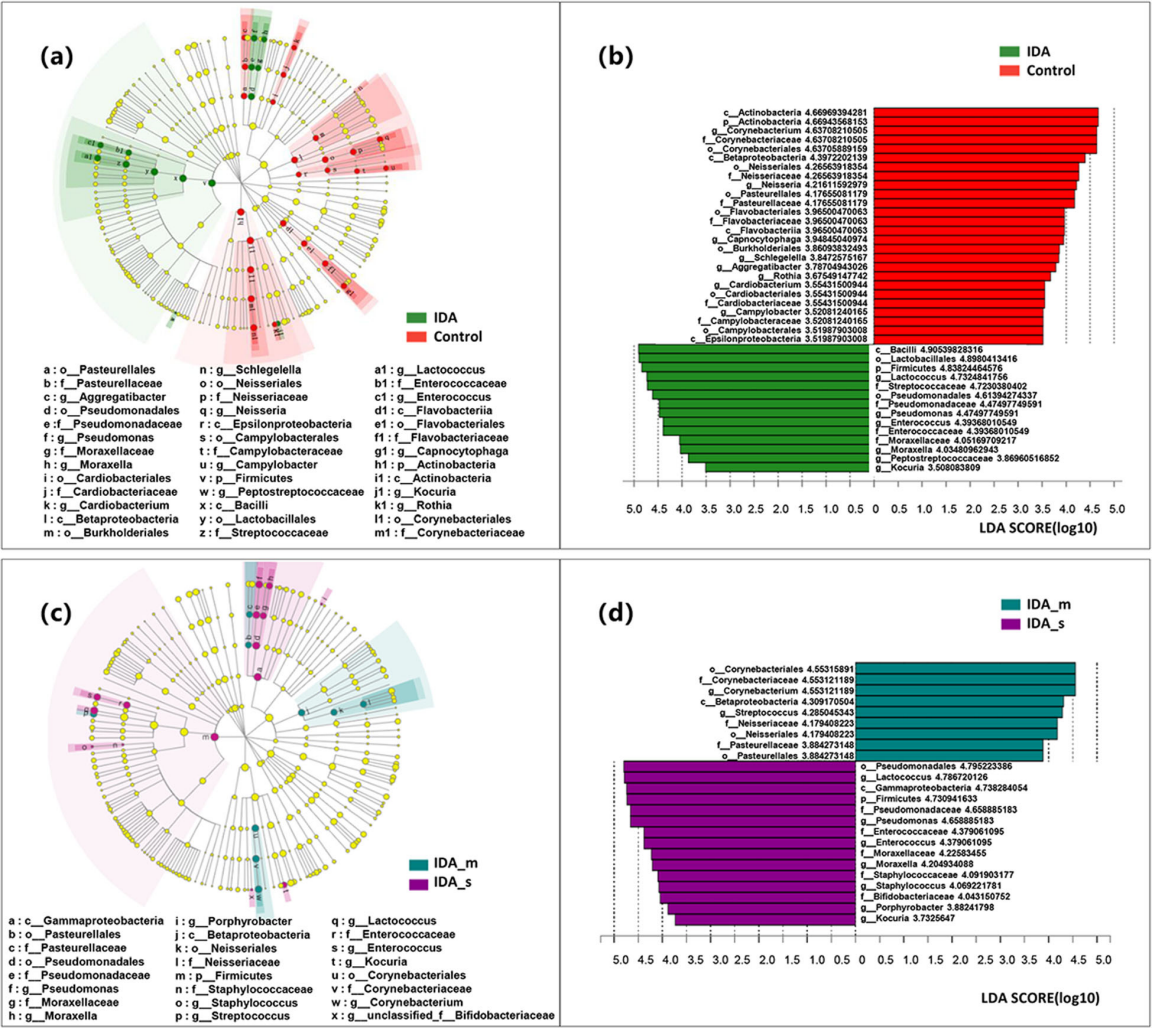
LEfSe analysis **a** The red and green dots represent the IDA-enriched taxa and the healthy control-enriched taxa, respectively. Concentric circles from the inside represent the taxonomic level of genus, followed by family, order, class, and phylum. **b** The red and green bars represent the LDA value of IDA-enriched taxa and of healthy control-enriched taxa, respectively (threshold value: LDA 3.5). **c** The purple and green dots represent the IDA_s-enriched taxa and the IDA_m-enriched taxa, respectively. **d** The purple and green bars represent the LDA value of IDA_s-enriched taxa and of h IDA_m-enriched taxa, respectively (threshold value: LDA 3.5)

Furthermore, the linear discriminant analysis effect size (LEfSe) was carried out. LEfSe considers biological relevance as well as statistical significance, and the larger LDA value indicates the taxa contribute more to the overall differences between the two groups. In this experiment, we selected the difference discrimination of phylum, class, order, family, and genus (threshold value: LDA 3.5). The LEfSe analysis revealed 42 discriminative features between the IDA and control groups (Fig. 5a, b) and 25 features between the IDA_s and IDA_m groups (Fig. 5c, d), with key phylotypes identified as microbiological markers. The taxa with increased abundance in the IDA and IDA_s groups showed significant evolutionary similarity, mainly belonging to the order Lactobacillales and Pseudomonadales.

#### Relationships of the bacterial profiles with haematologic parameters

**Fig. 6:**
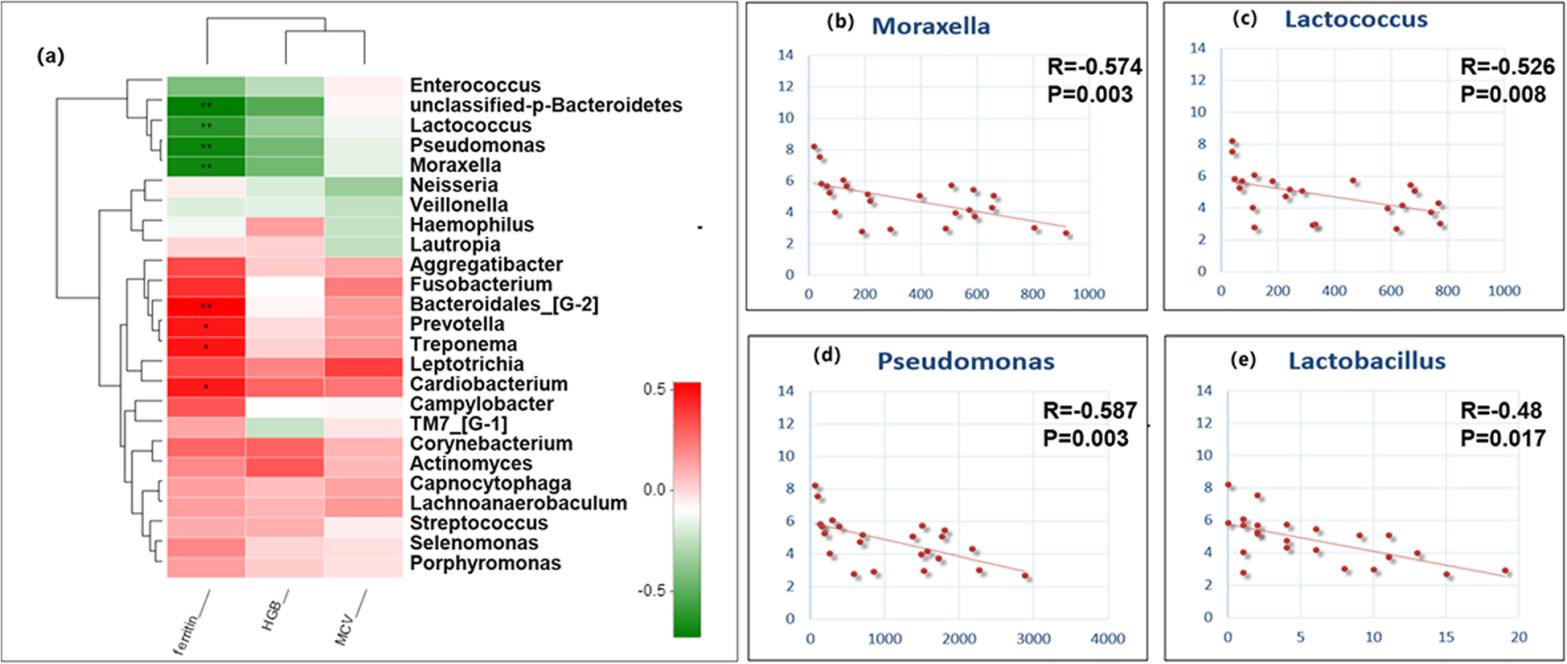
Significant correlations between the relative abundance of each genus and haematologic parameters. **a** Serum concentrations of ferritin, Hb (haemoglobin) and MCV (Mean Corpuscular Volume). * 0.01 < *P* ≤ 0.05, ** 0.001 < *P* ≤ 0.01. Significant correlations between serum ferritin and the relative abundances of Moraxella (**b**), Lactococcus (**c**), Pseudomonas (**d**) and Lactobacillus (**e**)

The Pearson coefficient was used to evaluate the correlation between the haematologic parameters (ferritin, Hb and MCV) and the relative abundance of each taxon (Fig. 6a). There were statistically negative correlations between the relative abundances of some genera (Moraxella, Lactococcus, Pseudomonas and, Lactobacillus) (Fig. 6b, c, d, e) and the serum ferritin level, while the genera Bacteroidales [G-2], Treponema, Prevotella, and Cardiobacterium exhibited strong positive correlations with the concentration of serum ferritin. The correlations between taxa abundance and concentration of haemoglobin or mean corpuscular volume (MCV) showed similar but less significant correlations (Table 4).

**Table 4:** Details of genera with high correlations with the three variables

	Hb_R	Hb_P	MCV_R	MCV_P	ferritin_R	ferritin_P
Actinomyces	0.365	0.079	0.14	0.515	0.248	0.243
Aggregatibacter	0.097	0.652	0.176	0.41	0.397	0.055
Bacteroidales_[G-2]	−0.002	0.993	0.214	0.316	0.564	0.004 ^**^
Campylobacter	−0.017	0.937	−0.007	0.973	0.369	0.076
Capnocytophaga	0.129	0.549	0.191	0.37	0.194	0.363
Cardiobacterium	0.331	0.115	0.294	0.163	0.507	0.012^*^
Corynebacterium	0.339	0.105	0.148	0.491	0.329	0.116
Enterococcus	−0.184	0.39	0.013	0.952	−0.325	0.121
Haemophilus	0.198	0.355	−0.156	0.467	−0.055	0.798
Lachnoanaerobaculum	0.147	0.492	0.212	0.319	0.199	0.352
Lactococcus	−0.273	0.196	−0.059	0.784	−0.526	0.008 ^**^
Lautropia	0.084	0.695	−0.169	0.431	0.07	0.744
Leptotrichia	0.258	0.223	0.422	0.04^*^	0.401	0.052
Moraxella	−0.348	0.096	−0.087	0.686	−0.574	0.003 ^**^
Neisseria	−0.113	0.599	−0.253	0.233	0.02	0.926
Porphyromonas	0.094	0.661	0.048	0.823	0.199	0.351
Prevotella	0.057	0.79	0.213	0.318	0.514	0.01 ^**^
Pseudomonas	−0.347	0.097	−0.087	0.686	−0.587	0.003 ^**^
Selenomonas	0.075	0.727	0.053	0.807	0.253	0.233
Streptococcus	0.166	0.437	0.018	0.934	0.169	0.43
TM7_[G-1]	−0.155	0.469	0.039	0.857	0.181	0.397
Treponema	0.079	0.713	0.226	0.289	0.517	0.01^**^
unclassified_p__Bacteroidetes	−0.415	0.044^*^	−0.003	0.99	−0.612	0.001 ^**^
Veillonella	−0.091	0.672	−0.164	0.443	−0.102	0.636

^*^ 0.01 < *P* ≤ 0.05, ^**^ 0.001 < *P* ≤ 0.01

## Discussion

This cross-sectional study enrolled 24 primary iron-deficiency anaemia (IDA) patients from West China Hospital, Sichuan University and 24 healthy controls. The genera Lactococcus, Enterococcus, Moraxella, Lactobacillus and Pseudomonas accounted for a higher proportion in the IDA communities and showed a statistically negative correlation with the concentration of serum ferritin.

There are two reasons for choosing the IDA population as an in vivo model. First, studies have pointed out that the concentrations of serum ferritin, saliva ferritin, and saliva lactoferrin have been pairwise positively correlated. During iron deficiency, significantly decreased concentrations of serum ferritin and haemoglobin lead to decreased iron availability for oral flora [23–26]. Second, the high prevalence of this disease makes it easy to obtain the required sample data. Diagnosis of IDA is defined with 2 of 3 abnormal blood tests: haemoglobin (< 110 g/L); serum ferritin (< 12 ng/L) and MCV (< 80 fL). Both groups were free from specific oral or systemic diseases to avoid undesired effects on oral microecosystem.

Illumina MiSeq sequencing revealed the profound impact of iron deficiency in vivo on modulating oral bacterial composition. The two groups shared the same dominant taxa (> 1% of the total DNA sequences). In good agreement with other studies, the phyla Firmicutes, Proteobacteria, Actinobacteria, Bacteroidetes, Fusobacteria, Spirochaetes, and Saccharibacteria_TM7 dominated the oral microbiota. In vivo, iron deficiency shifted the microbial population towards the orders Lactobacillales and Pseudomonadales. At the genus level, the abundances of Corynebacterium, Neisseria, Cardiobacterium, Capnocytophaga, and Aggregatibacter were significantly higher in healthy controls, while genera Lactococcus, Enterococcus, Lactobacillus, Pseudomonas and Moraxella showed higher proportions in the IDA group (*P* < 0.05). The abundance of the genera Pseudomonas, Moraxella, Lactococcus and Lactobacillus showed strong negative correlations with the serum concentration of ferritin (*P* < 0.05), while the genera Bacteroidales [G-2], Treponema, Prevotella, and Cardiobacterium exhibited strong positive correlations with the serum concentration of ferritin. No significant correlation was found between the relative taxa abundance and the concentrations of haemoglobin and MCV. It is not surprising because among various haematological parameters, serum ferritin has been defined as the most sensitive and reliable indicator of IDA, and it is directly related to whole-body iron storage [19]. The results were different from our previous in vitro study, in which haemolytic bacterial species such as α-haemolytic *Gemella* spp*., Streptococcus* spp. *and Granulicatella* spp. dominated the oral microbiota with the addition of iron chelator Bipy.

These phenomena may occur for the following three reasons.

First, elemental iron comes from different types of in vivo and in vitro sources. The nutrients of oral bacteria mainly come from saliva and gingival crevicular fluids [19]. Similar to other body fluids, saliva and gingival crevicular fluid provide iron in the forms of lactoferrin, transferrin, ferritin, haemoglobin and very little free iron [26]. In SHI medium, the addition of 2,2-bipyridyl and sheep blood removed free ferric and ferrous ions, leaving haemoglobin as the remaining source of iron [9]. Thus, haemolytic bacteria with the ability to destroy erythrocytes and obtain intracellular iron dominated. However, in the oral cavities of the IDA population, all iron sources were reduced, while some bacteria with increased abundance have a stronger iron harvesting ability by utilizing glycoproteins, especially lactoferrin, from the host and other microbes. Lactoferrin is a high-efficiency iron-binding protein and is more abundant in mucosal secretions and saliva. Lactoferrin can inhibit the growth and reproduction of a large number of bacteria with its high-efficiency iron-binding capacity, while *Enterococcus faecalis, Pseudomonas fluorescens, Lactobacillus* and *Bifidobacterium* strains are highly resistant to lactoferrin [30–33]. *Enterococcus* spp. have evolved a variety of mechanisms to obtain elemental iron. In the case of iron deficiency, *Enterococcus* spp. could not only utilize bivalent and trivalent free iron ions but also promote the degradation of host lactoferrin and transferrin [34, 35]. *E. faecalis* secreted highly efficient siderophores to capture the iron ions bound to the siderophores of Enterobacteria, Actinomycetes, Streptococcus, Staphylococcus, etc. and to inhibit the growth and toxicity of other bacteria. Some strains of Enterococcus can even exchange siderophores in low-iron situations [36]. Pseudomonas and Moraxella species are also known for encoding varieties of outer membrane siderophore receptors as well as component transferrin and lactoferrin receptors [37–39]. *P. aeruginosa* produces pyocyanin, a special iron carrier with the isohydroxamic acid group of gram-positive bacterial iron carriers and the catecholamine group of gram-negative bacterial iron carriers, that can inhibit the growth of other pathogenic bacteria [40–43]. *P. fluorescens* produces a group of special siderophores, pyoverdines, that play a role in biofilm formation and in the synthesis of acute virulence factors [40]. Therefore, these strains able to utilize a very wide range of iron complexes may have advantages in the oral environment of IDA patients.

Second, Lactobacillus are unusual organisms with a low requirement for iron, leading to advantages in iron-deficient environments. It was found in 1989 that the addition of iron-chelating agent in the culture medium could inhibit the growth of Enterobacteriaceae rather than that of Lactobacillaceae. Under iron-overload conditions, Lactobacillus is rapidly outcompeted with the faster growth velocity of other bacteria. Further radiological examination revealed that the iron content in several lactobacillus bacteria was extremely low, and manganese ions were capable of replacing iron ions in some metalloenzymes, covering almost all basic functions that iron usually accomplishes [18, 43]. In aerobic metabolism, this replacement sometimes acted as a possible defence method against oxygen toxicity [44].

Finally, some bacteria with increased abundance produce lactic acid, which promotes iron absorption. The bacterial clade consisting of Enterococcus, Lactococcus and Lactobacillus includes many genera of lactic acid bacteria from the phylum Firmicutes. The production of lactic acid reduced the environmental pH and promoted the transformation of ferric iron to ferrous iron, greatly increasing the solubility of iron [45]. Lactic acid can bind iron ions, forming a stable ferric lactate complex. Ferric lactate is less stable than ferritin in the host and can be utilized by ferritin of bacteria [46]. Furthermore, lactic acid can catalyse lipid peroxidation, resulting in partial ferritin dissociation and the release of free iron ions [47]. Thus, Iron deficiency shifted the microbial structure towards bacterial species more resistant to iron starvation conditions and/or those more capable of utilizing transferrin and lactoferrin rather than haem and haemoglobin.

As a part of dental plaque, oral bacteria can enter the bloodstream, causing bacteraemia through daily habits or after dental procedures. Historically, antibiotics were commonly recommended to prevent IE before dental procedures. Amoxicillin or ampicillin was proposed as the first choice. It has been reported that Enterococcus, Pseudomonas, Moraxella, Lactococcus and Lactobacillus can also lead to IE. Oral enterococci, Pseudomonas spp. and Moraxella catarrhalis are known for their high antibiotic resistance rate [8, 48, 49]. Enterococci have intrinsic resistance to β-lactams and cephalosporins via low-affinity penicillin-binding proteins, to aminoglycosides via the ability to block aminoglycosides from entering the cell wall, and to lincosamides and streptogramins via the ability to absorb folic acid from the environment [50]. As IE caused by enterococci accounts for 10% of the prevalence of this disease (the proportion is still rising), eradication requires prolonged administration and combinations of multiple drugs [8]. Pseudomonas spp. have intrinsic resistance to antibiotics such as ampicillin, amoxicillin, tetracycline, aminoglycosides and vancomycin [51, 52]. The preferred regimen for IE caused by *P. aeruginosa* is high-dose, extended-spectrum tobramycin combined with ceftazidime or cefepime [53]. Thus, in iron-deficient conditions, more precise use of antibiotics may need to be carried out with different populations before risky oral procedures.

However, there’s still the need for future study to analyze the microbiota at the level of species which can tell the more detailed microbial changes as well as investigate the microbiota after treatment to see if the microbiota would be restored.

## Conclusions

In summary, we demonstrated that iron deficiency in vivo conditions changed the oral microbiota community structure. Individuals with IDA, compared with healthy controls, displayed decreased overall bacterial diversity and altered taxonomic composition. The relative abundances of the genera Lactococcus, Enterococcus, Pseudomonas and Moraxella showed higher proportions in the IDA group, whose abundance was also statistically and negatively correlated with the concentration of serum ferritin (*P* < 0.05).

In contrast to our previous in vitro study, no significant differences were observed in the proportion of oral streptococci, which is the main pathogen of IE. This study cannot identify whether IDA can raise the risk of IE. However, as the genera that showed higher proportions in the IDA group were frequently reported as penicillin-resistant, the traditional choice of prophylactic penicillin may be inappropriate. The results showing disproportionate oral microbiota indicate more precise use of antibiotics may need to be carried out with different populations before risky oral procedures. This study offers new ideas of accurate and personalized prevention for IE, which may be the subject of further studies to uncover more information.

## Supplementary information

**Additional file 1: Table S1.** Clinical data of IDA group. **Table S2.** Comparison of bacterial taxonomy of samples between control and IDA groups on phylum and genus level.

## Data Availability

The datasets used and analyzed in the current study are available from the corresponding author upon reasonable request. The raw reads were then uploaded to the NCBI Sequence Read Archive (SRA) database (SRA number: SUB4333399).

## Abbreviations

ESC: European Society of Cardiology
Hb: Haemoglobin
HOMD: Human oral microbiome database
IDA: Iron-deficiency anaemia
IE: Infective endocarditis
LEfSe: Linear discriminant analysis effect size
MCV: Mean corpuscular volume
OTU: Operational taxonomical unit
PCoA: Principal coordinates analysis
