# Supplementary material for: Comparative analysis of the oral microbiota between iron-deficiency anaemia (IDA) patients and healthy individuals by high-throughput sequencing
Source: BMC Oral Health. 2019 Nov 21;19:255. doi: 10.1186/s12903-019-0947-6 (PMC6873577; doi:10.1186/s12903-019-0947-6)
Supplement: Supplementary file 1 — Additional file 1: Table S1. Clinical data of IDA group. Table S2. Comparison of bacterial taxonomy of samples between control and IDA groups on phylum and genus level. [file 12903_2019_947_MOESM1_ESM.docx]

| Table S1. Clinical data of IDA group   \| Sample \| Age range \| Weight \| Hb \| MCV \| Ferritin \| group \| \| --- \| --- \| --- \| --- \| --- \| --- \| --- \| \| IDA_1 \| 18-20 \| 48 \| 58 \| 75 \| 1.2 \| IDA_s \| \| IDA_15 \| 20-30 \| 55 \| 60 \| 76.2 \| 5.27 \| IDA_s \| \| IDA_5 \| 40-50 \| 70 \| 65 \| 75 \| 3.05 \| IDA_s \| \| IDA_16 \| 30-40 \| 52 \| 69 \| 76.8 \| 5.46 \| IDA_s \| \| IDA_7 \| 20-30 \| 60 \| 71 \| 60.6 \| 4 \| IDA_s \| \| IDA_19 \| 30-40 \| 46 \| 74 \| 85.4 \| 5.76 \| IDA_s \| \| IDA_8 \| 20-30 \| 65 \| 75 \| 60 \| 2.94 \| IDA_s \| \| IDA_3 \| 20-30 \| 57 \| 77 \| 67.1 \| 2.79 \| IDA_s \| \| IDA_6 \| 20-30 \| 62 \| 77 \| 68 \| 3.76 \| IDA_s \| \| IDA_11 \| 20-30 \| 50 \| 81 \| 64.6 \| 2.7 \| IDA_s \| \| IDA_20 \| 40-50 \| 55 \| 83 \| 65.1 \| 5.84 \| IDA_m \| \| IDA_12 \| 20-30 \| 51 \| 84 \| 65.7 \| 4.76 \| IDA_m \| \| IDA_17 \| 20-30 \| 50 \| 84 \| 69.1 \| 5.71 \| IDA_m \| \| IDA_4 \| 20-30 \| 52 \| 87 \| 64.3 \| 4.05 \| IDA_m \| \| IDA_2 \| 20-30 \| 55 \| 88 \| 70.9 \| 1.1 \| IDA_m \| \| IDA_9 \| 20-30 \| 59 \| 92 \| 74.4 \| 4.18 \| IDA_m \| \| IDA_13 \| 30-40 \| 47 \| 96 \| 69 \| 5.1 \| IDA_m \| \| IDA_14 \| 30-40 \| 47 \| 99 \| 84.3 \| 5.17 \| IDA_m \| \| IDA_24 \| 20-30 \| 59 \| 99 \| 86.7 \| 11.7 \| IDA_m \| \| IDA_22 \| 30-40 \| 50 \| 100 \| 73.7 \| 7.59 \| IDA_m \| \| IDA_18 \| 30-40 \| 55 \| 102 \| 67.6 \| 5.72 \| IDA_m \| \| IDA_21 \| 20-30 \| 55 \| 104 \| 82.8 \| 6.09 \| IDA_m \| \| IDA_23 \| 20-30 \| 60 \| 113 \| 74.8 \| 8.22 \| IDA_m \| \| IDA_10 \| 20-30 \| 58 \| 119 \| 81.3 \| 4.3 \| IDA_m \|   Hb (haemoglobin) MCV (Mean Corpuscular Volume).  Table S2. Comparison of bacterial taxonomy of samples between control and IDA groups on phylum and genus level | | | | | | | | |
| --- | --- | --- | --- | --- | --- | --- | --- | --- | --- | --- | --- | --- | --- | --- | --- | --- | --- | --- | --- | --- | --- | --- | --- | --- | --- | --- | --- | --- | --- | --- | --- | --- | --- | --- | --- | --- | --- | --- | --- | --- | --- | --- | --- | --- | --- | --- | --- | --- | --- | --- | --- | --- | --- | --- | --- | --- | --- | --- | --- | --- | --- | --- | --- | --- | --- | --- | --- | --- | --- | --- | --- | --- | --- | --- | --- | --- | --- | --- | --- | --- | --- | --- | --- | --- | --- | --- | --- | --- | --- | --- | --- | --- | --- | --- | --- | --- | --- | --- | --- | --- | --- | --- | --- | --- | --- | --- | --- | --- | --- | --- | --- | --- | --- | --- | --- | --- | --- | --- | --- | --- | --- | --- | --- | --- | --- | --- | --- | --- | --- | --- | --- | --- | --- | --- | --- | --- | --- | --- | --- | --- | --- | --- | --- | --- | --- | --- | --- | --- | --- | --- | --- | --- | --- | --- | --- | --- | --- | --- | --- | --- | --- | --- | --- | --- | --- | --- | --- | --- | --- | --- | --- | --- | --- | --- | --- | --- | --- | --- | --- | --- | --- | --- | --- |
| Phylum  name | Control-Mean(%) | | Control-Sd(%) | IDA-Mean(%) | IDA-  Sd(%) | P  value | Q  value |  |
| Firmicutes | 23.38 | | 8.676 | 37.49 | 14.4 | 0.000553 | 0.006633** |  |
| Proteobacteria | 25.5 | | 11.88 | 24.82 | 9.672 | 0.9589 | 0.9714 |  |
| Actinobacteria | 25.49 | | 12.26 | 16.88 | 13.67 | 0.01025 | 0.04924* |  |
| Bacteroidetes | 13.98 | | 6.588 | 12.09 | 6.694 | 0.3025 | 0.6422 |  |
| Fusobacteria | 8.722 | | 6.911 | 6.269 | 5.039 | 0.1768 | 0.5303 |  |
| TM7 | 1.616 | | 1.62 | 1.473 | 1.574 | 0.9507 | 0.9714 |  |
| Genus  name | Control-Mean(%) | | Control-Sd(%) | IDA-Mean(%) | IDA-  Sd(%) | P  value | Q  value |  |
| Corynebacterium | 18.5 | | 11.6 | 10.15 | 11.84 | 0.005205 | 0.05622 |  |
| Lactococcus | 4.951 | | 5.361 | 15.94 | 12.39 | 0.000321 | 0.01038* |  |
| Pseudomonas | 4.373 | | 5.218 | 10.66 | 8.357 | 0.001912 | 0.02581* |  |
| Streptococcus | 6.414 | | 3.402 | 6.077 | 3.461 | 0.7029 | 0.8759 |  |
| Leptotrichia | 6.28 | | 6.106 | 3.609 | 2.877 | 0.1195 | 0.4209 |  |
| Neisseria | 6.217 | | 6.309 | 3.062 | 4.403 | 0.006289 | 0.06298 |  |
| Actinomyces | 4.648 | | 1.958 | 4.457 | 4.686 | 0.2011 | 0.4721 |  |
| Capnocytophaga | 5.494 | | 2.896 | 3.577 | 3.071 | 0.02395 | 0.1552 |  |
| Enterococcus | 1.465 | | 1.783 | 6.392 | 5.544 | 5.55E-05 | 0.007054** |  |
| Prevotella | 3.454 | | 2.676 | 3.938 | 3.801 | 0.869 | 0.9844 |  |
| Selenomonas | 3.384 | | 3.378 | 2.237 | 2.596 | 0.127 | 0.4217 |  |
| Fusobacterium | 2.443 | | 1.88 | 2.659 | 2.637 | 0.8609 | 0.9821 |  |
| Moraxella | 1.383 | | 1.669 | 3.616 | 2.93 | 0.001663 | 0.02581* |  |
| Haemophilus | 3.121 | | 4.109 | 1.498 | 1.805 | 0.1146 | 0.4188 |  |
| Lactobacillus | 0.09293 | | 0.1054 | 0.2985 | 0.2375 | 0.000486 | 0.01312* |  |
| Cardiobacterium | 1.49 | | 0.7518 | 0.7592 | 0.6697 | 0.00144 | 0.02581* |  |
| Kocuria | 0.4227 | | 0.5351 | 1.1 | 0.8813 | 0.0016 | 0.02581* |  |
| unclassified_f__  Peptostreptococcaceae | | 0.01199 | 0.0168 | 0.07494 | 0.07759 | 0.001704 | 0.02581* |  |
| Bergeyella | 0.5087 | | 0.3253 | 0.2758 | 0.121 | 0.007317 | 0.06585 |  |
| Aggregatibacter | 2.784 | | 2.676 | 1.44 | 2.157 | 0.007812 | 0.06661 |  |
| Kingella | 0.5203 | | 0.5445 | 0.2158 | 0.3145 | 0.009536 | 0.07724 |  |
| Campylobacter | 1.605 | | 0.9416 | 0.9494 | 0.7456 | 0.01056 | 0.08147 |  |
| Kluyvera | 0.1135 | | 0.1561 | 0.2394 | 0.2032 | 0.0111 | 0.08175 |  |
| Eikenella | 0.2479 | | 0.2096 | 0.1156 | 0.1153 | 0.01444 | 0.1017 |  |
| Capnocytophaga | 5.494 | | 2.896 | 3.577 | 3.071 | 0.02395 | 0.1552 |  |
| Acinetobacter | 0.1066 | | 0.0751 | 0.2043 | 0.1605 | 0.02506 | 0.1562 |  |

Q value: corrected P value; correction method: FDR; * 0.01 < P < 0.05，** 0.001 < P ≤ 0.01，*** P ≤ 0.001.
